# Strategies to integrate community-based traditional and complementary healthcare systems into mainstream HIV prevention programs in resource-limited settings

**DOI:** 10.1186/s12992-018-0383-4

**Published:** 2018-07-04

**Authors:** Subash Thapa, Arja R. Aro

**Affiliations:** 1 Research Unit of General Practice, Department of Public Health, University of Southern Denmark, Odense, Denmark; 2 Unit for Health Promotion Research, University of Southern Denmark, Esbjerg, Denmark

**Keywords:** Community-based approaches, Complementary medicine, HIV prevention, Nepal, Traditional medicine

## Abstract

**Background:**

Global spending for HIV prevention has been decreasing over the years. As a result, several low-income countries, including Nepal, are increasingly facing the challenge to minimize the funding gap to continue providing HIV prevention services to the people. In this paper, we have attempted to clarify why it is important to integrate community-based traditional and complementary healthcare systems and mobilize them into the mainstream HIV programs to ensure access to HIV prevention messages, HIV testing, and treatment in resource-limited settings.

**Main body:**

First, we argue that the traditional and complementary healthcare practitioners can be mobilized to routinely provide HIV prevention messages to their clients, and, next, some of them can be trained to build their capacity to work as counselors or educators for HIV prevention in the community.

**Conclusion:**

These approaches, if implemented, can help continue HIV prevention initiatives and contain the HIV epidemic at the local level in the rural communities with limited cost and resources.

## Background

HIV testing, the disclosure of HIV positive status and antiretroviral treatment (ART) uptake are important strategies for the HIV prevention. However, more than 40% of population at-risk of HIV worldwide do not have an access to HIV testing services and more than 50% of the people living with HIV do not receive ART [1]. Global spending for HIV prevention has been decreasing over the years and several low-income countries are increasingly facing the challenge to minimize the funding gap to continue providing HIV prevention services [2, 3]. Even, to maintain the current progress in HIV, it is estimated that low-income countries need to significantly increase their investment from US$20 billion to more than US$35 billion by 2031 [4].

In Nepal, HIV is a concentrated epidemic among at-risk population groups. The epidemic is mostly driven by heterosexual transmission, which accounts for more than 85% of total new HIV infections [5]. Among the total population infected with HIV, 40% are the general male population and 35% are the general female population. The other population groups include injecting drug users (8%), transgender people (2%), their clients (5%), men having sex with men (9%), and female sex workers (1%) [5]. The Nepal Demographic Health Survey of 2016 has reported that 89% of men and 80% of women have never been tested for HIV. And, only 12% of men and 19% of women have knowledge about the place to access ART [6]. Due to the reduction in funding over the years, the Nepalese health system is encountering problems in continuing HIV prevention services, especially in the rural areas. As a result, the health system is less likely to meet the demand of the beneficiaries’ related to HIV prevention due to the lack of materials and supplies at the local level. Thus, HIV researchers and program managers meanwhile are more interested in knowing the impact of funding problems, especially in relation to HIV prevention.

Funding gaps and resource limitations may threaten to derail the HIV response. For instance, Nkonki and colleagues have noted that the prevention of mother-to-child transmission intervention in South Africa did not significantly increase HIV testing and treatment uptake because of non-availability of health workers and lack of HIV test kits [7]. Unlike HIV, the uncontrolled burden of other infectious diseases, such as plague and tuberculosis, had been experienced in the history of medicine when public health initiatives were let down because of resource-related reasons. In Nepal, poor political will and limited domestic funding may favor interventions that do not specifically aim to reach the vulnerable populations. As a result, people may have limited access to HIV services or they may face delays in the uptake of health services, including HIV testing and treatment. The absence of health service or limited access to it, especially in the rural areas, may enable people to continue engaging in traditional and complementary healthcare practices [8]. And, if the interventions to integrate and mobilize the traditional and complementary healthcare systems into the mainstream health system are not in place, this can exacerbate HIV stigma, non-disclosure of HIV status, and delays in healthcare access, which may eventually lead to higher transmission and mortality rates and, particularly, the poorest and most vulnerable will be affected the most [9].

The current HIV prevention programs or interventions should adopt or incorporate approaches to continue increasing people’s access to HIV testing and ART services. We argue that a strategy to collaborate with or mobilize the community-based traditional healthcare systems and networks in the mainstream health systems could be crucial to ensure that there is equitable access to effective HIV prevention messages, HIV testing, and treatment services for all those who need it [10]. Therefore, in this paper, we illuminate the reasons why it is important to mobilize community-based traditional and complementary healthcare systems into mainstream HIV programs to ensure access to HIV prevention messages, HIV testing and treatment, and contain the HIV epidemic in the rural communities with limited cost and resources in Nepal.

### Community-based traditional and complementary healthcare in HIV prevention

Traditional medicine is the total sum of the knowledge, skills, and practices based on the theories, beliefs, and experiences indigenous to different cultures, whether explicable or not, used in the maintenance of health as well as in the prevention, diagnosis, improvement or treatment of physical and mental illness [11]. The term ‘complementary medicine’, which is also used interchangeably with traditional medicine, refers to a broad set of healthcare practices that are not fully integrated into the dominant healthcare system. The World Health Organization (WHO) estimates that in a low-income country like-Nepal, 80% of the population may engage in a group of traditional and complementary healthcare practices, such as Faith healing, Energy Healing, Yoga, Herbal medicine, Ayurveda, and Homeopathy, in combination with or in place of the mainstream HIV programs [11]. The WHO emphasizes to integrate the community-based traditional and complementary healthcare systems within the mainstream healthcare system to increase people’s access to healthcare.

Nepal has a long history of practicing traditional and complementary medicine, which has been passed on from generation to generation through community-based belief systems. Recognizing this fact, the Constitution of Nepal along with the National Health Policy-2014 call for the promotion of traditional Ayurveda medicine, naturopathy, homeopathy and other complementary healthcare systems in the country [5]. The Ayurveda and Alternative Medicine Unit are institutionalized under the Ministry of Health and Population, which formulates policies and guidelines, and manages Ayurveda and other traditional healthcare systems. There are altogether two Ayurveda hospitals, 14 zonal Ayurveda dispensaries, 61 district Ayurveda health centers and 305 Ayurveda dispensaries, and in average, 1.2 million client consultations for Ayurveda treatment for general health problems in these institutions last year. [5] It is believed that the clear majority of the population, including people living with HIV or the vulnerable population groups, seek help from traditional and complementary medicine in the rural areas of Nepal [9].

Despite this high patronage, the mainstream health system often ignores community-based traditional healthcare beliefs and practices, which has been evident as one of the reasons for low uptake of HIV testing and ART services in the rural areas of several low-income countries, including Nepal [9, 12, 13]. We may need to look back and realize that even in the period of huge investments on HIV prevention, the traditional and complementary medicine systems have been practiced by a huge majority of population groups, and today and in the future, we can assume that this will definitely continue. We believe, in terms of designing and developing cost-effective approaches for HIV prevention, the traditional and complementary healthcare systems and the mainstream healthcare system are not completely incompatible and can be complementary.

### Strategies to integrate traditional and complementary health care into the mainstream HIV prevention programs

In agreement with what Flint has stated, we believe that, to increase access to prevention messages, HIV testing, and treatment services in resources-limited settings, there needs to be explicit recognition of, and further strategies to integrate and mobilize the traditional and complementary healthcare systems and practitioners within mainstream HIV prevention interventions [10]. Moreover, integrating traditional and complementary healthcare systems into HIV prevention programs can help make a shift from a vertical disease-specific approach to an integrated and comprehensive response to increasing access to HIV prevention [14]. A comprehensive approach to HIV prevention may generally help to provide HIV prevention services to the wider population groups in the community [10].

One general strategy of integration is to encourage traditional and complementary medicine practitioners to incorporate HIV prevention messages into their routine practices and increase their clients’ general awareness on HIV prevention. For instance, traditional beliefs and practices continue to significantly influence the way in which HIV is understood in the community, and through this strategy, we can make a beneficial impact on making people aware about HIV testing and ART. At the local level, this strategy can further help people change their attitudes towards HIV and HIV prevention, and behave normally with the people living with HIV [15]. One of the other strategies is that, we can adapt the concept of ‘medical pluralism’, in which all the practitioners from Yoga, Ayurveda, Homeopathy and Indigenous medicine systems can be alternately mobilized to routinely identify the clients possessing HIV-related risk behaviors and to provide HIV-related prevention messages and the information about the closest health institutions offering HIV prevention services [10, 16]. After all, the success of the HIV prevention interventions in a resource-limited setting can be seen as the best use of health services by the people who need them the most. This can significantly increase the efficiency of the interventions to provide services at a lower cost.

The most empirically tested strategy is engaging the traditional and complementary healthcare practitioners into the mainstream HIV prevention programs via training and building their capacity to serve as counselors or health educators in the community [10, 16–18]. For example, working in Nepal, Poudel and colleagues (2005) have reported that, after providing training to indigenous healers, they were able to provide culturally acceptable health education to the community people, and played a role in reducing HIV stigma via visiting the people living with HIV in their homes [17]. The community-based health practitioners are noted to have historical knowledge of community-based indigenous systems, and those who are trained to provide HIV-related services have been proven to play a major role in HIV prevention because of their accessibility and acceptability in the community [10, 16].

The way this strategy works is that, when we mobilize the local traditional and complementary healthcare practitioners as educators and counsellors, a small portion of the community may already start adopting HIV prevention behaviors. For instance, via everyday encounters and interactions, the family members and the neighbors of these practitioners would be the first among all the community members who will be motivated for changing their risk behaviors. In addition, the trained practitioners may professionally and personally help the other community members to access HIV testing services and discuss the test result, help the people with positive test results to cope with their HIV diagnosis, increase their feeling of control, and track the improvement in HIV treatment outcomes. Moreover, we believe, through this strategy, an emphasis can be given to help local health practitioners taking leading roles in programs, and building their capacity to manage and lead their own projects at the local level. Simultaneously, it can also create an environment for behavioral interventions to be easily accepted in the community [19].

Tilburt and Franklin have noted that the mobilization of traditional and complementary medicine practitioners at the local level can also help solve some issues related to medical ethics [20]. On one hand, the mainstream health system can ethically acknowledge the traditional and complementary healthcare systems, accommodate diverse healing practices, and try to generate scientific evidence related to community-based systems. On the other hand, it also makes sure that the traditional and complementary healthcare practitioner do practice in agreement with ethical guidelines and based on scientific evidence [11]. For example, working in South Africa, Joanne Wreford has noted that, drawing traditional and complementary healthcare practitioners into prevention and treatment interventions in a spirit of constructive engagement and mutual respect can challenge existing stereotypes and foster cooperative relationships [21]. Given mutual respect, traditional and complementary healthcare practitioners can be successfully drawn into biomedical prevention and treatment interventions, and thereby improve their efficacy [21]. However, one should also keep in mind that it is only possible with enthusiasm, research-based evidence and building the capacity to integrate and mobilize the traditional and complementary healthcare practitioners in HIV prevention in Nepal.

## Conclusion

There needs to be explicit recognition of, and further strategies to collaborate with the traditional and complementary healthcare systems and practitioners, and mobilize them in the mainstream HIV programs to increase people’s access to prevention messages, HIV testing and treatment services. Two of the strategies for collaboration and partnership are that: general HIV prevention messages are to be delivered by the traditional and complementary healthcare practitioners in their routine practices, and some of the practitioners can be trained to build their capacity to work as counselors or educators in the field of HIV prevention in the community. Additionally, development and reformation of the traditional and complementary healthcare systems and their integration with mainstream healthcare system should be well informed through qualitative and mixed-methods research in Nepal.

## Abbreviations

ART: Antiretroviral treatment
HIV: Human Immunodeficiency Virus
WHO: World Health Organization
